# Biomimetic Magnetic Nanocarriers Drive Choline Kinase Alpha Inhibitor inside Cancer Cells for Combined Chemo-Hyperthermia Therapy

**DOI:** 10.3390/pharmaceutics11080408

**Published:** 2019-08-12

**Authors:** Ylenia Jabalera, Alberto Sola-Leyva, Ana Peigneux, Federica Vurro, Guillermo R. Iglesias, Jesus Vilchez-Garcia, Inmaculada Pérez-Prieto, Francisco J. Aguilar-Troyano, Luisa C. López-Cara, María P. Carrasco-Jiménez, Concepcion Jimenez-Lopez

**Affiliations:** 1 Department of Microbiology, Faculty of Sciences, University of Granada, 18071 Granada, Spain; 2 Department of Biochemistry and Molecular Biology I, Faculty of Sciences, University of Granada, 18071 Granada, Spain; 3 Department of Neurosciences, Biomedicine and Movement Sciences, Anatomy and Histology Section, University of Verona, 37134 Verona, Italy; 4 Department of Applied Physics, Faculty of Sciences, University of Granada, 18071 Granada, Spain; 5 Department of Pharmaceutical and Organic Chemistry, Faculty of Pharmacy, Campus of Cartuja, 18071 Granada, Spain

**Keywords:** biomimetic, magnetic nanoparticle, ChoKα1 inhibitor, Ff35, drug delivery, hyperthermia

## Abstract

Choline kinase α1 (ChoKα1) has become an excellent antitumor target. Among all the inhibitors synthetized, the new compound Ff35 shows an excellent capacity to inhibit ChoKα1 activity. However, soluble Ff35 is also capable of inhibiting choline uptake, making the inhibitor not selective for ChoKα1. In this study, we designed a new protocol with the aim of disentangling whether the Ff35 biological action is due to the inhibition of the enzyme and/or to the choline uptake. Moreover, we offer an alternative to avoid the inhibition of choline uptake caused by Ff35, since the coupling of Ff35 to novel biomimetic magnetic nanoparticles (BMNPs) allows it to enter the cell through endocytosis without interacting with the choline transporter. This opens the possibility of a clinical use of Ff35. Our results indicate that Ff35-BMNPs nanoassemblies increase the selectivity of Ff35 and have an antiproliferative effect. Also, we demonstrate the effectiveness of the tandem Ff35-BMNPs and hyperthermia.

## 1. Introduction

**Figure 1:**
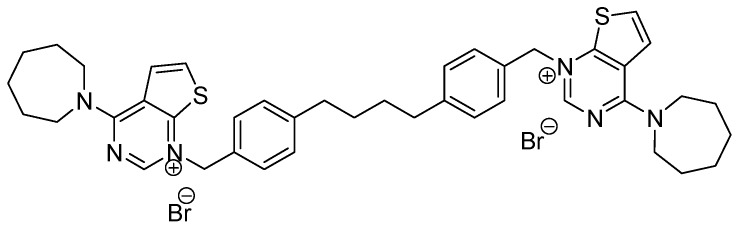
Chemical structure of choline kinase α 1 (ChoKα1) inhibitor Ff35.

Chemoresistance to cancer is a major concern [1,2] that has prompted the development of new effective therapies. Among then, those that affect lipid metabolism, whose alteration occurs in many types of cancer [3,4], have gained great attention. Particularly, the metabolism of choline has been associated with tumor onset and progression. In this context, choline kinase α 1 (ChoKα1) isoform has been considered as biomarker of tumor progression and carcinogenesis [5,6,7], and it has emerged as one of the most promising therapeutic target enzymes in cancer. ChoK catalyzes the phosphorylation of choline to generate phosphocholine which, through cytidine 5´-diphosphocholine (CDP-choline) [8], produces phosphatidylcholine. ChoKα also regulates the mitogen-activated protein kinase (MAPK) and phosphoinositide 3-kinase (PI3K/AKT) signaling pathways [9]. Extensive efforts have been made to synthesize and improve ChoKα1 inhibitors in order to use them as chemotherapeutic agents [10,11], since it is well known that ChoKα1 inhibitors have antiproliferative effect on several tumor cell lines [12]. Hemicolinium-3 was the first one used in clinics, but it has multiple side effects, mainly due to the inhibition of choline uptake and the impediment of the synthesis of acetylcholine [13]. Afterward, bis-pyridinium and bis-quinolinium derivatives, namely MN58b and RSM-932A, respectively, were also produced with high capacity to inhibit ChoKα1 activity and cell proliferation [14], and, even more recently, several other compounds have been synthesized based on the chemical structure of the former compounds [12]. In particular one of them, here referred as Ff35 (Figure 1), contains bioisoteric rings of the quinoline, where carbon atoms have been replaced by nitrogen and sulphur atoms, and the same spacer as that of the compound MN58b [15]. These changes would lead, on one hand, to an increase of the polarity (and solubility) of the compound and, on the other hand, to an increase of the specificity of the drug. The latter is due to the increasing number of free electron pairs that these modifications induce, which can form hydrogen bonds or dipole–dipole type junctions between the molecule and the enzyme, thus increasing the antiproliferative effect of the drug.

However, it has been described that certain inhibitors of ChoKα1, besides hemicholinium-3, are also capable of inhibiting choline uptake, thus making it more difficult, if not impossible, for their use in systemic clinical treatments [16]. For this reason, firstly, for any newly synthesized inhibitor of ChoKα1 and before proposing it as antitumoral drug, it is imperative to design protocols to discern whether the resulting inhibition of tumor cell proliferation is due to the inhibition of the enzyme (which would allow the potential use of the compound in clinics) or whether it is caused by the inhibition of the choline uptake (which may prevent its potential use). Moreover, if the latter is the case, it is also crucial to design strategies to avoid the inhibition of choline uptake. A possibility to prevent such inhibition is to use carriers to introduce the specific ChoKα1 inhibitor in the cell without interacting with the choline transporters. In this context, one alternative is to couple the compound with nanoparticles that could enter the cell by endocytosis, as we propose for the first time in the present study.

Among the potential nanocarriers, magnetic nanoparticles are preferred because of their biocompatibility and their magnetic properties, which, on one hand, allow their guidance to the target site and their concentration therein by the application of external magnetic fields and, on the other, they allow the nanoparticle itself to be used as hyperthermia agent [17]. Biomimetic magnetic nanoparticles, BMNPs, mediated by *Magnetococcus marinus* MC-1 magnetosome membrane protein MamC, have demonstrated themselves to be promising nanocarriers, able to couple with drugs forming stable nanoassemblies at physiological pH, while efficiently releasing the drug in acidic (tumor) environments in response to pH changes. In fact, these BMNPs present novel features compared to other nanoparticles produced inorganically and/or other biomimetic nanoparticles [18]: (1) They are superparamagnetic (single magnetic domain) at room and body temperature, behaving as nonmagnetic in the absence of an external magnetic field (thus minimizing aggregation), while they present a large magnetic moment per particle under the influence of an external magnetic field, thus optimizing their guidance and concentration at the target site. Such behavior is due to the fact that these BMNPs are larger than most commercial and/or other biomimetic magnetic nanoparticles. (2) the BMNPs are cytocompatible and even more, (3) they are produced by means of an eco-friendly, cost effective, easily scalable method.

To our understanding, this is the first study that explores the functionalization of BMNPs with a ChoKα1 inhibitor, such as Ff35, with the goal of obtaining a potential nanoassembly suitable for a targeted chemotherapy that avoids possible side effects related to the inhibition of the choline uptake. Moreover, the simultaneous use of hyperthermia is explored to optimize the effect of the targeted chemotherapy in terms of increasing the cytotoxic effect of the nanoassembly. The *in vitro* antitumor activity and the internalization of these complexes were investigated in the human hepatoblastoma cell line, HepG2.

## 2. Materials and Methods

### 2.1. Expression and Purification of MamC. Synthesis and Characterization of the BMNPs by Transmission Electron Microscop

MamC expression and purification was performed as previously described by Valverde-Tercedor et al. [19]. *Escherichia coli* TOP10 (Invitrogen, Grand Island, NY, USA) were transformed with the pTrcHis-TOPO plasmid (Invitrogen, Grand Island, NY, USA) carrying the MamC protein coding gene (Mmc1_2265). These cells were grown at 37 °C and MamC overproduction was induced with isopropyl-1-thio-β-d-galactopyranoside (IPTG). Afterwards, a HiTrap chelating HP column (Ge Healthcare, Chicago, IL, USA) in an ÄKTA Prime Plus FPLC System (GE Healthcare, Chicago, IL, USA) was used to purify the protein under denaturing conditions (6 M urea). Lastly, dialysis was performed for a gradual removal of urea, which allowed MamC to refold progressively and the purity was evaluated by SDS-PAGE electrophoresis.

BMNPs were synthesized from the following master solution: 10 μg mL^−1^ of the recombinant MamC, Fe(ClO_4_)_2_ (2.78 mM), NaHCO_3_/Na_2_CO_3_ (3.5 mM/3.5 mM), FeCl_3_ (5.56 mM), pH 9, elaborated from oxygen-free stock concentrated solutions of the individual compounds [20]. The master solution was incubated for 30 days in an anaerobic COY chamber at 25 °C and 1 atm total pressure. After this time, BMNPs were concentrated by using a magnet and the supernatant was discarded. Subsequently, the precipitates were washed three times with Milly-Q deoxygenated water, suspended in HEPES (4-(2-hydroxyethyl)-1-piperazineethanesulfonic acid) buffered saline solution (0.01 M HEPES, pH 7.2, 0.15 M NaCl), and sterilized at 121 °C for 21 min in an autoclave before their use.

The BMNPs used in the present study have been previously characterized in García-Rubia et al. [18], and, therefore, only basic mineral characterization is included in the present manuscript. According to these results, the BMNPs used in the present study are superparamagnetic magnetic nanoparticles at 300 K, composed of ~95 wt% of magnetite and ~5 wt% of MamC, with an isoelectric point of 4.4 and specific surface area of 90 m^2^/g.

For transmission electron microscopy (TEM) analyses of the BMNPs, a STEM Philips Model CM20 microscope was used. BMNPs were embedded into Embed 812 resin and then, ultrathin sections (50–70 nm) were cut with a Reichert Ultracut S microtome (Leica Microsystems GmbH, Wetzlar, Germany). ImageJ 1.47 software was used to measure particle sizes on multiple micrographs with over 1000 nanoparticles measured for each experiment to ensure reproducibility.

Hysteresis cycles and zero field cooling-field cooling (ZFC-FC) curves were carried out by using a superconducting quantum interference device (SQUID) 5 T magnetometer (Quantum Design MPMS XL, San Diego, CA, USA). Blocking temperature (T_B_) was calculated as the temperature at which the maximum in magnetization occurred in ZFC curve.

### 2.2. Formation of Ff35-BMNPs Nanoassemblies

The kinetics of Ff35 adsorption on the magnetic nanoparticles was studied to determine the time required for this adsorption to reach equilibrium. In these experiments, an aliquot of 5 mg of BMNPs was mixed with 1 mL of Ff35 100 µM in HEPES buffer (10 mM HEPES, 150 mM NaCl, pH 7.2) for several time intervals up to 48 h. After the incubation time, the Ff35-BMNPs nanoassemblies (here referred as Ff35-BMNPs) were collected with a magnet and washed twice with HEPES buffer. Then, the supernatants were measured by UV-Vis spectroscopy at a wavelength of 304 nm and these measurements were used to calculate the percentage of the absorbed compound. The molar absorptivity of Ff35 in HEPES buffer at 304 nm was determined as 2677.5 L mol^−1^ (*R*^2^ = 0.9991) from the slope of a standard calibration straight line. More than three replicas were performed per experiment. The standard deviation of the absorbance measurements was used to calculate the error in the concentration of Ff35 in the supernatant (*[Ff35]_sn_*).

The adsorption isotherm to calculate the saturation concentration of Ff35 adsorbed on the BMNPs was determined by mixing an aliquot of 5 mg of BMNPs with 1 mL of several concentrations of Ff35 (ranging from 0 up to 200 µM) in HEPES buffer. These preparations were incubated at 25 °C under continuous stirring for 6 h (time needed to reach equilibrium, according to the kinetics experiments). Three replicas were performed per each measurement. After the incubation time, supernatants were harvested, particles were washed, and UV-Vis spectroscopy measurements of all supernatants were performed as described above. The data were fitted to the model of Langmuir-Freundlich (LF) by using Origin 8. The LF model is based on Equation (1), where (*Q*) is the amount of adsorbed drug per mass unit of adsorbent, (*C_e_*) is the amount of nonadsorbed compound, (*K_LF_*) is the LF affinity constant, and (*r*) is the cooperativity coefficient. A value of *r* < 1 means a negative cooperativity whereas a value of *r* > 1 means a positive cooperativity [18,21].

(1) $$Q=\frac{Q_{max}{\left(K_{LF}C_{e}\right)}^{r}}{1\;+\;{\left(K_{LF}C_{e}\right)}^{r}}.$$

### 2.3. Cell Culture

The human hepatoblastoma HepG2 cell line, used in this work, was acquired from the European Collection of Animal Cell Cultures (Salisbury, UK). The cells were grown in Minimum Essential Medium (MEM) containing 10% heat-inactivated fetal bovine serum (FBS) supplemented with 2 mM l-glutamine, 1% nonessential amino acids, 100 U mL^−1^ penicillin, and 100 µg mL^−1^ streptomycin in a humid atmosphere with 5% CO_2_ as 37 °C, and subcultured at a ratio of 1:10 once a week.

### 2.4. Inhibition of Choline Kinase α1 by Ff35

The effect of Ff35 on ChoK was assayed in purified ChoKα1 as previously described by Schiaffino et al. [12], by determining the rate of incorporation of ^14^C from [methyl-^14^C]choline into phosphocholine, both in the absence (control) or presence of different inhibitor concentrations. Briefly, the final reaction mixture contained 100 mM Tris-HCl (pH 8.5), 10 mM MgCl_2_, 10 mM ATP, and 20 ng of purified ChoKα1. The reaction was initiated with 1 mM [methyl-^14^C]choline (4500 dpm/nmol) and incubated at 37 °C for 10 min. The assay was stopped by immersing the reaction tubes in boiling water for 3 min. Aliquots of the reaction were applied to the origin of silica gel plates in the presence of phosphocholine (0.1 mg) and choline (0.1 mg) as carriers. The chromatography was developed in methanol/0.6% NaCl/28% NH_4_OH in water (50:50:5, *v*/*v*/*v*) as solvent, and phosphocholine was visualized under exposure to iodine vapour. The corresponding spot was scraped and transferred to scintillation vials for measurement of radioactivity by a Beckman 6000-TA (Madrid, Spain) liquid-scintillation counter. The 50% inhibitory concentrations (IC_50_ value) were determined from the % activity of the enzyme at different concentrations of synthetic inhibitor by using a sigmoidal dose-response curve (the ED50plus v1.0 software).

### 2.5. Cell Proliferation Assay

HepG2 cells were seeded onto 96-well plates (10000 cells/well) and grown in MEM/10% FBS for 24 h. After 24 h, the medium was removed and 100 μL of fresh medium containing Ff35 (1, 5, and 10 µM), BMNPs (300 µg mL^−1^), or Ff35-BMNPs (Ff35 1 μM and BMNPs 300 µg mL^−1^) were added for different times. Cell viability was assayed by the MTT (3-(4,5-dimethylthiazol-2-yl)-2,5-diphenyltetrazolium bromide). Formazan crystals were dissolved in 100 μL of DMSO, and the absorbance was read at a wavelength of 570 nm using a microplate reader (HTX Microplate Reader BioTek Instruments, Winooski, VT, USA). The GI_50_ (half-maximal growth inhibitory concentration) value was determined from the % cell viability at different concentrations of synthetic inhibitor by using a sigmoidal dose-response curve (the ED50plus v1.0 software), referencing this value to an untreated cells control taken as a 100% of the viability.

### 2.6. Inhibition of Choline Uptake by Ff35 and Ff35-BMNPs

Choline uptake was assayed as previously reported [22]. HepG2 cells were incubated for 10 min at 37 °C in a MEM/10% FBS medium containing soluble Ff35 (0.5 and 1 µM), BMNPs (300 µg mL^−1^), or Ff35-BMNPs (concentration of Ff35 was 0.5 μM and BMNPs was 150 µg mL^−1^ or that of Ff35 was 1 μM and BMNPs was 300 µg mL^−1^). After 10 min, 24 h, and 48 h of treatment, the cells were immediately exposed to a pulse of [methyl-^14^C]choline (16 μM, 31 Ci/mol, in well) for 5 min at 37 °C. The incorporation of choline was stopped by medium aspiration followed by two washes in ice-cold PBS containing 580 µM choline. Then, the cells were solubilized in NaOH 0.1 N and an aliquot was used to determine the total amount of radiolabeled choline taken up by the cells, measured by liquid scintillation using a Beckman 6000-TA counter (Madrid, Spain).

### 2.7. Internalization of BMNPs or Ff535-BMNPs in the Cells

HepG2 cells were grown in six-well dishes for 24 h. Then, BMNPs (300 µg mL^−1^) or Ff35-BMNPs (concentration of Ff35 was 1 μM and BMNPs was 300 µg mL^−1^) or only MEM/10% FBS medium, as a control, were added for 24 h. Cells were collected using trypsin and centrifuged at 1500 rpm for 5 min in MEM/10% FBS. Cell pellets were fixed in 2.5% glutaraldehyde and 2% paraformaldehyde in 0.05 M cacodylate buffer for 4 h at 4 °C. The samples were washed three times with cacodylate buffer and postfixed in an aqueous solution of 1% OsO_4_ containing 1% potassium ferrocyanide for 1 h at 4 °C in darkness. The following washes were done (25 °C): 0.15% tannic acid in cacodylate buffer, cacodylate buffer, and H_2_O. The samples were left in 2% uranyl acetate for 2 h and washed several times with H_2_O. Then, dehydration in ethanol solutions rising from 50% to 100% was done at 4 °C. The samples were embedded in resin (EMbed 812/100% ethanol (1/1)) for 60 min at room temperature, the same resin at a 2/1 ratio for 60 min, and then resin without ethanol overnight. For polymerization, the samples were incubated in pure resin for 48 h at 60 °C. Ultrafine sections (50–70 nm) were cut using a Leica Ultramicrotome R and contrasted using 1% aqueous uranyl acetate for 5 min and lead citrate in a CO_2_-depleted atmosphere for 4 min [23]. A Zeiss Libra Plus 120 electron microscope was used to visualize the sections.

### 2.8. Hyperthermia Analysis

Magnetic hyperthermia experiments were carried out using a laboratory-built AC current generator, based on a Royer-type oscillator. The AC source was connected to a double five-turn coil built with a copper tube 4 mm in diameter. This allowed control of the temperature of the coil by flowing thermostated water. The coil was 20 mm in diameter and 45 mm long. The magnetic field frequency was 197 ± 3 kHz, and its strength was *H* = 21 kA/m (*B* = 26.4 mT in air) at the center of the coil, where the samples were placed, measured with a NanoScience Laboratories Ltd., Probe (Newcastle, UK), with 10 µT resolution. All samples were previously prethermostated at 37 °C. Prior to any determination, the adiabatic condition of the system was verified by subjecting a sample of Milli-Q water as control, in order to ensure that any temperature changes in the samples under study were due to the action of the magnetic field, and not a consequence of environmental temperature gradient. A preliminary experiment was performed with a suspension of bare BMNPs (300 µg mL^−1^) to set the conditions (frequency and strength of the field and time of application) needed to guarantee that a temperature of 43 °C was reached. The sample temperature was determined with an optical fiber thermometer (Optocon AG, Dresden, Germany). For actual hyperthermia experiments, HepG2 cells were incubated for 24 h at 37 °C with either 300 µg mL^−1^ BMNPs or Ff35-functionalized BMNPs and exposed to the AC magnetic field for time lapses ranging between 1 and 3 h. Immediately after the hyperthermia treatment, the cells were processed for the MTT test.

### 2.9. Statistics

The results are shown as averages ± SEM. A one-way ANOVA was done with post hoc comparisons by Scheffé’s test (SPSS 13.0). *p* < 0.05 is considered statistically significant.

## 3. Results and Discussion

**Figure 2:**
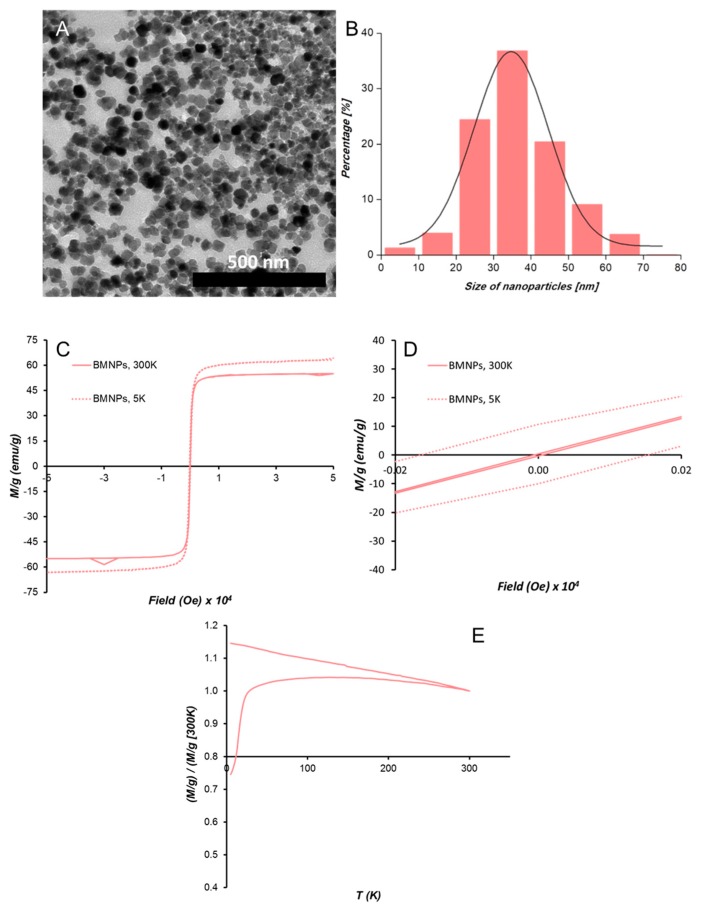
Biomimetic magnetic nanoparticles (BMNPs) characterization. (**A**) TEM image of BMNPs and (**B**) crystal size distribution. (**C**) Hysteresis cycle of BMNPs at 300 K and 5 K. (**D**) Detail of the hysteresis cycle in the absence of external magnetic field at 300 K and 5 K. (**E**) Zero field cooling-field cooling (ZFC-FC) curves of BMNPs.

BMNPs exhibited well-developed faces and a size ranging from 20 to 50 nm, with an average crystal size of 35 ± 8 nm, according to TEM analyses (Figure 2A,B). The hysteresis loop of BMNPs showed a typical ferromagnetic behavior at 5 K, while at 300 K, these nanoparticles showed zero coercivity, which indicates their superparamagnetic character, being that the magnetization saturation (Ms) was at 300 K 55 emu/g (Figure 2C,D). The ZFC-FC curves at 500 Oe show a slow increase in magnetization (Figure 2E). In fact, the T_B_ was 150 K for BMNPs. According to Prozorov et al. [17], this higher T_B_ and slow magnetization increase is related to particles that expose a large magnetic moment per particle and high crystallinity.

**Figure 3:**
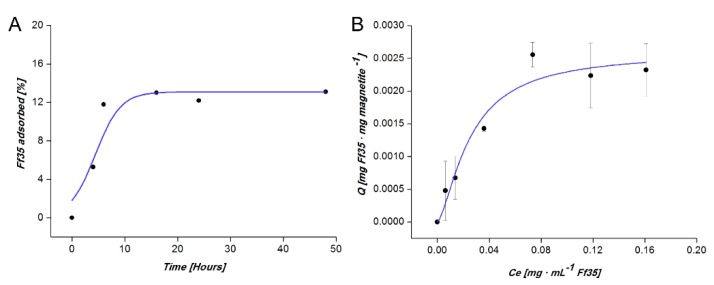
Adsorption kinetics (**A**) and adsorption isotherm (**B**) of Ff35 on BMNPs. The line represents the nonlineal weighted least-squares (NWLS) fitting of the experimental data according to LF model. In the case of adsorption kinetics, the vertical error bars are smaller than the symbol.

The kinetics of Ff35 adsorption on BMNPs over time shows that the system reached equilibrium at ~6 h (Figure 3A). The amount of adsorbed drug per amount of nanoparticles (*Q*) increased with the equilibrium concentration of Ff35 in the supernatant (*C*_e_) at a higher rate at the lowest C_e_ values. Such a rate decreased as *C*_e_ increased (Figure 3B). The adsorption isotherm adjusts to the Langmuir-Freundlich (LF) models (*R*^2^ = 0.93402), showing a drug loading capacity (*Q_max_*) of 0.0026 ± 0.0003 mg Ff35 mg magnetite^−1^. This model introduces the effects of energetic heterogeneity of the surface and the cooperativity between Ff35 molecules, meaning that once a Ff35 molecule is coupled to BMNPs, it lowers the energy required for the coupling of the next ones. The values of the LF affinity constant (*K_LF_*) and cooperativity coefficient (*r*) parameters, calculated by means of this model were of 40 ± 10 mg of Ff35 per mg of magnetite and 1.5 ± 0.6, respectively.

Our results show that BMNPs are able to carry 0.0026 mg of Ff35 per mg of magnetite (*Q_max_*). Due to the fact that this is the first study on Ff35 adsorption on magnetite nanoparticles (or on any other nanoparticles), comparisons of the *Q_max_* of the present study are done in reference to other studies involving either the BMNPs used here or other nanocarriers. The *Q_max_* value obtained in the present manuscript was lower than those values obtained for the coupling of other drugs like doxorubicin (DOXO) to BMNPs. For example, the adsorption of DOXO on the same BMNPs was 0.69 ± 0.03 mg DOXO/mg BMNPs [18] or 0.41 ± 0.03 mg DOXO/mg apatite for DOXO adsorption on citrate-coated apatite [24]. Even though *Q_max_* for Ff35 is about two orders of magnitude lower than that for other compounds, this amount that is coupled to the BMNPs is enough to have a cytotoxic effect as it will be shown below.

The BMNPs’ loading capacity could be explained, on the one hand, by the electrostatic interaction occurring at pH 7.2 (pH value at which the coupling occurs) between Ff35, which exposes two basic groups, positively charged and the negatively charged magnetite surface (isoelectric point (iep) = 4.4) [18], which allows Ff35 to be bound to the BMNPs, as previously demonstrated for other nanoassemblies involving BMNPs and positively charged molecules [18]. However, when the environmental pH decreases approaching the iep of the BMNPs (as it naturally does in a tumor environment, for instance, in the endosome-lysosome compartment [25]), the electrostatic interactions between Ff35 and BMNPs weaken, prompting Ff35 release. This characteristic presents a relevant point for the potential clinical application of Ff35-BMNPs nanoassemblies, since no Ff35 release is expected in the bloodstream until the nanoparticles reach the target tumoral (acidic) environment. On the other hand, the results show that not only electrostatic interactions between the drug and the BMNPs would be responsible for the Ff35-BMNPs nanoassemblies formation, since the r coefficient obtained in LF model (*r* > 1) demonstrates a strong positive cooperativity between the molecules of Ff35 during the adsorption process. This type of interaction has been previously described in DOXO adsorption on citrate-coated apatite nanocrystals [24] and on BMNPs [18].

**Figure 4:**
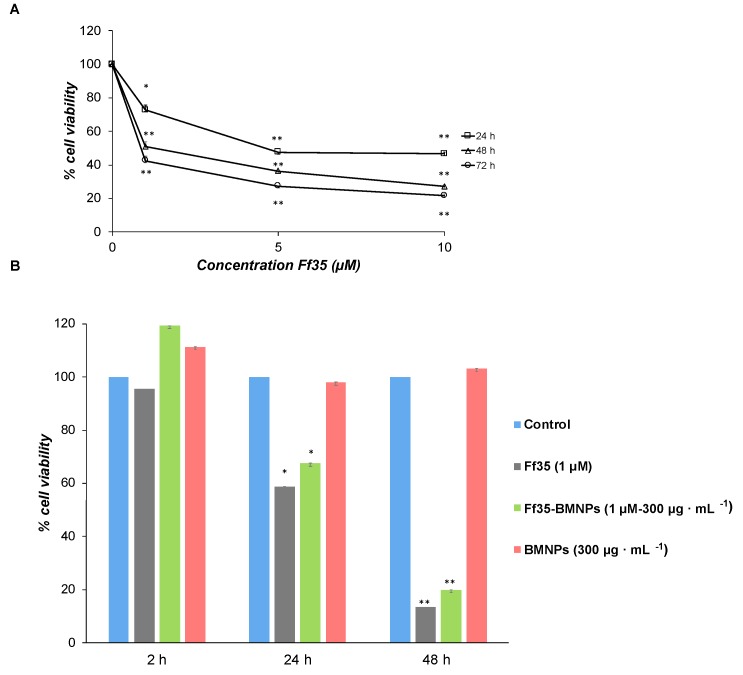
Effects of Ff35, BMNPs, and Ff35-BMNPs on HepG2 cell proliferation. HepG2 cells growing in the log phase were incubated with Minimum Essential Medium containing 10% heat-inactivated fetal bovine serum (MEM/10% FBS) in the presence or absence of Ff35 up to 10 μM (**A**) or Ff35 1 μM, BMNPs 300 µg mL^−1^ or Ff35-BMNPs (concentration of Ff35 was 1 μM and that of BMNPs was 300 µg mL^−1^) (**B**). Cell viability was determined by MTT assay and normalized to that of the respective control cells. These experiments were performed twice in triplicate. * *p* < 0.05, ** *p* < 0.001 compared to their respective controls.

Soluble Ff35 show a negative effect on HepG2 cell growth in a time and concentration-dependent manner (Figure 4A). The IC_50_ values derived from the growth inhibition curves were of 6.23 *±* 0.39, 1.37 *±* 0.004, and 0.45 *±* 0.10 μM for 24, 48, and 72 h, respectively. Lactate dehydrogenase (LDH) activity in the culture medium was not detected after any of the treatments up to 10 μM of Ff35 (data not shown), so the decrease in cell proliferation observed after the treatment with soluble Ff35 could not be attributed to any acute cytotoxicity produced by plasma-membrane leakage. Ff35-BMNPs also significantly decreased cell growth after 24 or 48 h of treatment. HepG2 cell proliferation was not affected by the presence of BMNPs, which demonstrates the cytocompatibility of the BMNPs.

**Figure 5:**
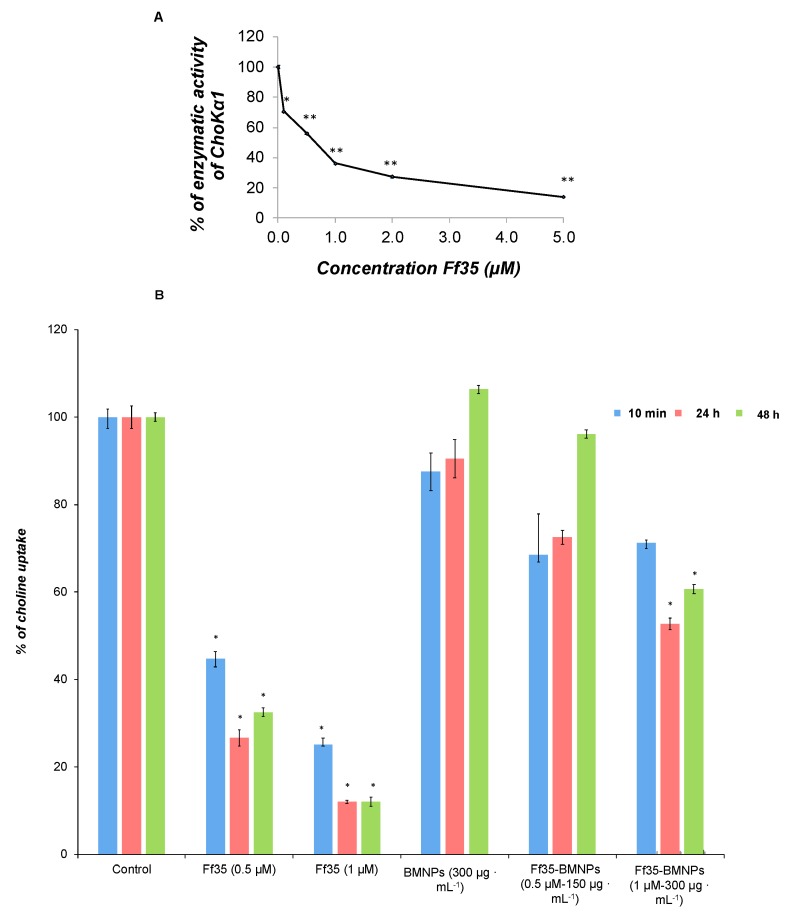
(**A**) Effects of Ff35 on ChoKα1 activity assayed in purified ChoKα1 exposed to different Ff35 concentrations. The results are expressed as percentage of enzymatic inhibition. (**B**) Choline uptake assay in HepG2 cells. Choline uptake was determined in cells treated for 10 min, 24 h, and 48 h soluble Ff35 or Ff35-BMNPs. Results are normalized to their respective control. These experiments were performed three times in triplicates. * *p* < 0.05, ** *p* < 0.001 compared to their respective controls.

Soluble Ff35 inhibits the activity of ChoKα1 (Figure 5A), with an IC_50_ value of 0.46 ± 0.079 μM. In addition, choline uptake was also inhibited in the presence of soluble Ff35 in 55% and 75% at 0.5 μM and 1 μM, respectively, after only 10 min of exposure. The effect was even more noticeable after 24 or 48 h of treatment, reaching inhibition levels of up to 68% and 88% at Ff35 0.5 μM and 1 μM, respectively, after 48 h of treatment (Figure 5B). These results clearly indicate that soluble Ff35 inhibits strongly both choline activity and absorption, and that the action of this compound in both processes is associated with a decrease in cell proliferation.

Interestingly, the treatment with Ff35-BMNPs for 10 min did not produce significant changes in the incorporation of choline into the cells compared with cells treated with BMNPs (Figure 5B) or in control cells (Figure 5B). After 24–48 h of treatment, only a significant inhibition of choline uptake was observed at a concentration of Ff35 1 μM and BMNPs 300 µg mL^−1^. Nevertheless, this inhibition was much lower than that observed with soluble Ff35 at the same concentration. The results indicate that the functionalization of BMNPs with Ff35 strongly reduces the inhibitory effect of Ff35 on choline uptake. To avoid completely the action of Ff35 on choline uptake, it would be necessary to control the concentration of Ff35 as well as the time of treatment. It is likely that after a long treatment there may be some release of the compound from the BMNPs that were not endocitated. This released Ff35 may be responsible for the slight inhibition observed during treatment with Ff35-BMNPs (Ff35-BMNPs 1 μM and BMNPs 300 µg mL^−1^). However, the amount of compound released from Ff35-BMNPs (Ff35-BMNPs 0.5 μM and BMNPs 150 µg mL^−1^) was not sufficient to inhibit choline uptake.

However, despite the fact that Ff35-BMNPs had little effect on the uptake of choline, it produced a marked decrease in cell proliferation, as mentioned above, similar to that observed in cells treated with soluble Ff35 at the same concentration (Figure 4B). This is very important because, by coupling Ff35 to the BMNPs, the antiproliferative effect of the compound is maintained as if Ff35 was soluble, whereas any secondary effects associated to the inhibition of choline uptake induced by soluble Ff35 are avoided or reduced. This new property improves the potential clinical use of Ff35 that would be otherwise impeded.

**Figure 6:**
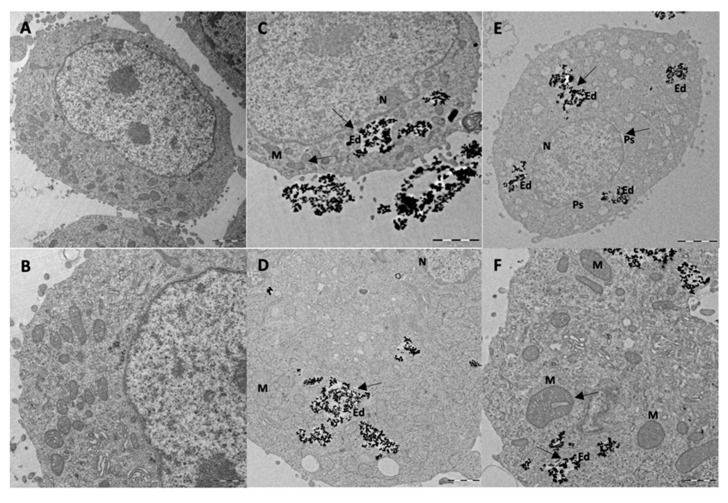
Internalization of BMNPs and Ff35-BMNPs. Ultrastructural alterations produced by Ff35-BMNPs. (**A**,**B**): Control HepG2 cells. (**C**,**D)**: HepG2 cell exposed to BMNPs has ultrastructure similar to control cells. (**E**,**F**): Treatment with Ff35-BMNPs (concentration of BMNPs was 300 µg mL^−1^ and Ff35 was 1 μM). Perinuclear space (Ps) is visible. The two nuclear membranes were separated showing a dilatation of the Ps. It is notably mitochondrial (M) rarefaction, disorganization, and dilatation. Both BMNPs and Ff35-BMNPs were internalized by HepG2 cells via endocytosis and compartmentalized in endosomes (Ed). Scale bar corresponds to 2 µm (A,C,E) and 1 µm (B,D,F).

TEM was used to visualize the internalization of BMNPs or Ff35-BMNPs (concentration of Ff35 was 1 μM and BMNPs was 300 µg mL^−1^), and to determine the possible morphological changes caused by Ff35-BMNPs exposure. Ultrastructural analysis by TEM showed that BMNPs were internalized in the cells by endocytosis (Figure 6). Control cells (Figure 6A,B) showed mitochondria having a dense matrix as well as many cisternae of rough endoplasmic reticulum (ER) well structured, indicating absence of cell damage. No ultrastructural cell damage was observed following BMNPs internalization until 24 h incubation (Figure 6C,D), confirming the cell biocompatibility of these nanoparticles at a concentration of 300 μg mL^−1^. Both the BMNPs (Figure 6C,D) and Ff35-BMNPs (Figure 6E,F) were internalized via endocytosis and sorted into endosomes. As it can be seen, cells treated with Ff35-BMNPs showed ultrastructural alteration, such as a notable dilatation of perinuclear space increasing separation of two nuclear membranes, loss of mitochondrial density, and an abnormal shape. In general, the cells exposed to the BMNPs showed a similar appearance to control cells, with not significant structural alterations. These experiments confirm the cell damage following upon Ff35-BMNPs observed above, and explain why, while soluble Ff35 inhibits choline uptake, Ff35-BMNPs do not. While soluble Ff35 requires choline transporter to enter the cell, Ff35-BMNPs enter the cells by endocytosis, and thus, *via* a method that does not depend on choline transporters. Again, these results open the possibility for a clinical use of ChoKα1 inhibitors by making their uptake by the cells independent of choline transporters. Moreover, the experiments of the present study provide a protocol that allows us to disentangle whether the cytotoxic activity of a ChoKα1 inhibitor such a Ff35 is due to the selective inhibition of the enzyme, or on the contrary, due to the nonselective inhibition of the choline uptake by the cell as an undesirable secondary effect. This protocol could be standardized and extended to other molecules.

**Figure 7:**
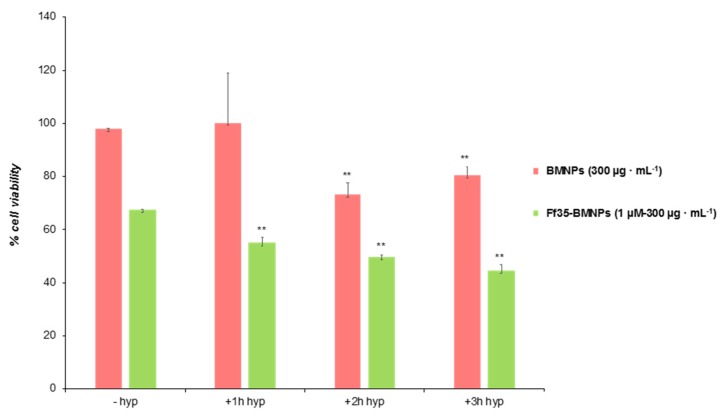
Effects of Ff35-BMNPs and hyperthermia on cell proliferation in HepG2 cells. HepG2 cells growing in log-phase were incubated with MEM/10% FBS in the absence or presence Ff35-BMNPs (concentration of Ff35 was 1 μM and BMNPs was 300 µg mL^−1^) for 24 h, and exposed to an alternative magnetic field for 1, 2, or 3 h. Cell number was determined by the MTT assay and expressed as percentage of control cells. Percentage of viability is normalized to that for the control cells. Results represent the mean ± SEM of three independent experiments conducted in triplicate. ** *p* < 0.001, when compared with their respective control values.

We consider now the effect of hyperthermia and the combination hyperthermia-Ff35 on cell proliferation. In all cases, the HepG2 cells were subjected to the magnetic field for periods of 1, 2, and 3 h. We assume that, according to generally accepted results, tumor cells are more sensitive than healthy ones to a temperature increase [26], and, although 43 °C is widely accepted as a common threshold for apoptosis of various cancer cells, many reports indicate that different thermal sensitivities exist among different cell lines [27]. Hence, preliminary experiments aimed at setting the optimal conditions of hyperthermia in HepG2 with the used BMNPs either bare or functionalized with Ff35. Figure 7 shows the effect of hyperthermia on the viability the cells, in comparison with cells not subjected to the magnetic field. Hyperthermia reinforced cytotoxicity on both cells treated with BMNPs and, much more so, on cells treated with Ff35-BMNPs. As it can be observed, after 2 h (and also 3 h) of hyperthermia regime there was a significant decrease in viability (73% after 2 h and 79% after 3 h) of cells maintained in the presence of BMNPs and AC field, revealing the cytotoxic effect of hyperthermia. However, there were not significant differences between these two different times. On other hand, significant differences in viability were found when the cells were treated with Ff35-BMNPs, with and without field. It is shown in Figure 7 that the viability of cells treated with Ff35-BMNPs without hyperthermia reached 67%, these values being lower in those cells treated with Ff35-BMNPs with hyperthermia (54.8% for 1 h, 49.7% for 2 h, and 44.5% for 3 h). Since BMNPs are cytocompatible, these results show that the temperature rise induced by them upon application of an alternating magnetic field was responsible for the reduction in cell viability. However, such reduction was more evident and faster upon the combination of drug release and hyperthermia. It is proposed that hyperthermia may not only increase the local temperature, but also trigger the release of Ff35 from the BMNPs in the cells, as already demonstrated to occur for other compounds [28].

Therefore, these results open the door, not only to the potential use of ChoKα1 inhibitors without the secondary effect linked to the inhibition of choline uptake, but also, to the application of a dual therapy based on targeted drug release and hyperthermia to potentiate the cytotoxic activity of these compounds on tumor cells.

## 4. Conclusions

In summary, this study shows that the coupling of the ChoKα1 inhibitor Ff35 to BMNPs offers great advantages. On one hand, since the nanocarrier is a magnetic nanoparticle, it opens the possibility of a magnetic guidance to the target site through the application of a continuous magnetic field. Also, it offers the possibility of also using the nanocarrier as a hyperthermia agent, thus combining the cytotoxic effect of the molecule with the cytotoxic effect induced by hyperthermia and the triggering of drug release from the BMNPs that hyperthermia induces. Finally, and no less important, it offers the potential of Ff35 entering the cell independently of the choline transporters. This is crucial, as the coupling of Ff35-BMNPs allows the compound to exert a cytotoxic activity comparable to that of the soluble compound while avoiding the secondary effect linked to the inhibition of choline uptake. Also, it provides a protocol that could be extensible to other molecules to disentangle the cytotoxic effect of the drug in terms on enzyme inhibition and/or inhibition of choline uptake. Therefore, the novel design of the nanoassemblies of Ff35-BMNPs showed in this study and the demonstration of the activity of such a compound without interfering in the choline uptake are crucial results, as they would allow the potential use of these ChoKα1 inhibitors as antitumoral drugs that would otherwise be compromised.
